# The current pandemic, a complex emergency? Mental health impact of the COVID-19 pandemic on highly vulnerable communities in Guatemala

**DOI:** 10.1177/00207640211027212

**Published:** 2022-11

**Authors:** Dana Alonzo, Marciana Popescu, Pinar Zubaroglu-Ioannides

**Affiliations:** 1 Graduate School of Social Service, Fordham University, West Harrison, NY, USA; 2 Department of Social Work, Suleyman Demirel University, Isparta, Turkey

**Keywords:** COVID-19, mental health, complex emergency, low-income, Guatemala

## Abstract

**Background:**

On March 5th, Guatemala declared a ‘State of Calamity’ in response to the COVID-19 pandemic and strict lockdown measures were initiated. The psychological consequences of these measures are yet to be fully understood. There is limited research on the psychological impact of the virus in the general population, and even less focused on Latin America and high-risk communities characterized by poverty, limited mental health resources, and high rates of stigma around mental illness. The goal of this study is to examine the psychological impact of COVID-19 across several highly vulnerable districts in Guatemala.

**Methods:**

A semi-structured phone interview was conducted of 295 individuals in multiple districts in Guatemala City to assess self-perceived mental health consequences related to the pandemic. Sociodemographic, medical, and mental health data were collected. Chisquares and *t*-tests used for categorical and continuous variables, as appropriate, to describe the sample. Binary logistic regressions were estimated to examine associations between sociodemographic characteristics and mental health symptoms (anxiety, stress, depression, burnout, escalation of pre-existing mental health symptoms, and a sense of safety).

**Results:**

The results indicate high levels of anxiety and stress in all target communities. Significant differences based on gender, age, and the number of children in the household were identified: women and older adults experience higher rates of stress and anxiety associated with the pandemic; while families with greater number of children experience higher levels of burnout.

**Conclusion:**

Contextualizing the current pandemic as a complex emergency can help inform further studies focusing on socioeconomic challenges and higher vulnerabilities as preconditions affecting the impact of the pandemic on mental health. Given the limited available resources for mental health care in Guatemala, informal networks of care may play an important role in meeting the needs of those individuals experiencing increased psychological distress resulting from the pandemic.

## Introduction

Guatemala has experienced a rate of COVID-19 much higher than other Central American countries (Ministry of Health, 2020). According to the Ministry of Heath, as of November, 2020, there have been 111,262 confirmed cases of COVID-19 in Guatemala, with 3,821 deaths (Latin American News Dispatch, 2020). On March 5th, a ‘State of Calamity’ was declared and strict lockdown and curfew measures and travel restrictions were initiated thereafter to reduce the spread of the virus (Ministry of Health, 2020; US Department of State, 2020). A nationwide nightly curfew was instituted between the hours of 16:00 and 04:00. During curfew hours, individuals were required to stay at home and those in breach of the curfew were subject to a fine or imprisonment. Only essential personnel, including police, private security, medical professionals, and food-delivery driver were exempt. Outside of curfew hours, all individuals were mandated to comply with social distancing rules, requiring people to stay at least 5 ft apart and utilize facemasks in all public spaces. Failure to comply with these regulations could result in heavy fines. All land, sea, and air borders were closed, and entry to most non-Guatemalan nationals was barred, with only diplomatic, health, and security personnel, as well as exceptional cases designated by the government, exempt from the closure. International and domestic flights were suspended and public transport was limited to operate at a 50% capacity (GardaWorld, 2020). In addition, there were reports that some local Guatemalan communities were taking unofficial action to restrict individuals from entering or exiting their communities, referred to as ‘Blockades’ (https://gt.usembassy.gov). In some cases, non-Guatemalan citizens were prevented from entering or leaving a community or, upon leaving were not allowed to return (https://gt.usembassy.gov). These restrictions were extended every several weeks until October, 2020.

Further complicating the pandemic-related stressors in Guatemala are the pre-pandemic conditions of the country. Over the past 10 years, Guatemala experienced some economic stability, leading to moderate growth rates, but not translating in significant poverty reduction. According to The World Bank (2020a), Guatemala is the fifth poorest economy in Latin America and the Caribbean (LAC), maintain high rates of poverty and inequality. Its slow economic progress is particularly impended by poor fiscal health, challenges with the judicial system, and limited labor freedom (Index of Economic Freedom, 2020). In regards to manifestations of extreme poverty, Guatemala has the sixth highest rate of malnutrition in the world and the highest in LAC, with over 60% of the population being food insecure (The World Bank, 2020a). Almost half (47%) of all children under five suffer of chronic malnutrition (The World Bank, 2020a), with malnutrition rates being higher amongst children in indigenous communities (58%) and in lowest income quintile communities (66%). Rampant gang violence, and high rates of drug trafficking create significant insecurity among poor Guatemalan communities, known as ‘Red Zone Districts (RZDs)’ within Guatemala City. These communities have experienced rates of poverty, crime (Human Rights Watch, 2020; Overseas Security Advisory Council [OSAC], 2019), and community and gender violence (Gerkin, 2020; Wilson, 2020) at highest levels in Latin America and among the highest in the world (Ogrodnik & Borzutzky, 2011). One study using a community survey (Puac-Polanco et al., 2015) found that one in five participants have experienced serious violent events, and violence was significantly associated with subsequent mental health problems especially for vulnerable groups such as women, indigenous groups, and urban Guatemalans. Prevalence estimates of 40.7% for depression, 23.3% for alcohol-related disorders, and 50% for PTSD have been reported in Guatemala (Branas et al., 2013). Rates of substance use, gang violence (including, extortions, and drug trafficking), domestic violence (Mercier, 2020), and teen pregnancy are also high (OSAC, 2019; Schwartz, 2020).

Compounding these issues is the scarcity of mental health resources in Guatemala, especially in the poorer communities (Godoy-Paiz, 2005), with less than 1% of the country’s general health budget devoted to mental health care (Duarte & Martinez, 2015; Rodríguez et al., 2002) and no existing national policies providing protections for individuals struggling with mental illness (WHO, 2014). The few organized mental health services that do exist in Guatemala are often inadequate, located in the city center limiting access to those in the provinces where needs are the highest, and are financially inaccessible (Godoy-Paiz, 2005). Added to this, the country (like many other countries in the region) has a fragile, precarious safety net, with highly vulnerable communities experiencing the absence of effective welfare policies and programs, at much higher rates during the pandemic (Dupraz-Dobias, 2020; Rauls, 2020).

Extended periods of quarantine imposed during previous public health crises (i.e, SARS), such as those imposed in Guatemala, have been shown to be associated with separation, isolation, boredom and sense of uncertainty, depression, symptoms of PTSD, and in some cases even suicide (Bai et al., 2004; Brooks et al., 2020; Chatterjee & Chauhan, 2020). Longer periods of quarantine are associated with greater severity of symptoms (Brooks et al., 2020; Hawryluck et al., 2004). Other studies have demonstrated that quarantines result in a sense of feeling trapped and a perception of loss of control (Chatterjee & Chauhan, 2020). A recent review of psychological outcomes related to periods of quarantine found numerous negative outcomes, including stress, depression, irritability, insomnia, fear, confusion, anger, frustration, boredom, and stigma, some of which persisted even after the quarantine was lifted. Stressors most often related to these outcomes included greater duration of confinement, having inadequate resources to meet basic needs, difficulty securing medical care and medications, and financial hardships (Brooks et al., 2020).

Research examining the mental health impact of the current COVID-19 pandemic and ensuing quarantine periods have largely focused on three areas: (1) those infected with the virus; (2) frontline healthcare providers; and (3) far behind, a small but growing body of research examines the mental health consequences of quarantine in the general population. In Spain, for example, a cross-sectional survey of adults found that between 20% and 30% of respondents expressed clinical range scores on measures of anxiety and depression, and psychological stress was reported by approximately 48% of respondents (Odriozola-González et al., 2020). Similarly, Roma et al. (2020), found elevated rates of stress and depression among adults during quarantine in Italy. An online survey of mood disturbance among adults in Australia found elevated scores for tension, depression, anger, fatigue, and confusion, and below average scores for vigor (Terry et al., 2020). A survey of adults in Ecuador, one of the few studies in Latin America examining the mental health impact of COVID-19 in the general population, found a significant number of respondents reported severe or extremely severe levels of depression, anxiety, and stress (Tusev et al., 2020). In India, a survey of the general population found that participants reported feeling helpless, depressed, anxious, and experienced mental fatigue, insomnia, dissatisfaction, and disconnected from others (Kochhar et al., 2020). Literature on frontline workers confirms the even greater levels of stress, depression, irritability, insomnia, fear, confusion, anger, and frustration than in the general population (Brooks et al., 2020; Pfefferbaum & North, 2020).

Pre-existing conditions are a source of great concern for spread of infection and increased vulnerability to negative outcomes of extended quarantine. When considering the factors that serve as a vulnerability for negative mental health consequences, research to date has largely emphasized the role of pre-existing conditions in two areas: (1) physical health status, and (2) pre-existing mental health conditions. Few, if any, studies have explored the mezzo level factors characteristic of the RZDs in Guatemala and common across many countries in Latin America, for their potential to serve as pre-existing vulnerabilities that might increase the likelihood of the pandemic resulting in significant mental health impairment.

Mezzo level factors from an ecological perspective are commonly defined as socioeconomic and sociocultural factors experienced at the community level by families, groups, neighborhoods and organizations that impact one’s ability for adaptive functioning (Bronfenbrenner, 1977; Eriksson et al., 2018). These factors may be considered within a Social Determinants of Health framework (SDH). The SDH framework can contribute to a better understanding of health inequalities within and across populations and support to the design of clinical interventions and public health policies. Further, the social determinants of behavioral health impact not only quality-of-life outcomes, such as socioeconomic status, educational attainment, access to healthy and affordable food choices, employment and job stability, housing status, exposure to toxic environments, but also access to quality health and behavioral health services (Baffour, 2017). According to the Commission on Social Determinants of Health (CSDH) of the World Health Organization (WHO), health inequalities and inequities result from the contexts within which people grow, live, work, and age, and the systems that exist to deal with illness (WHO, 2008). Socially predetermined forces contribute to create these contexts that essentially determine who will live and what their quality of life may be. Within communities like the RZDs, for example, monetary loss is a major stressor during and post periods of quarantine as employment is interrupted or people are unable to work entirely. Financial loss due to quarantine is associated with severe socio-economic distress and is a contributing factor for anger and anxiety (Kochhar et al., 2020; Mihashi et al., 2009; Pellecchia et al., 2015). Food insecurity is another huge problem plaguing RZDs. Nutritional factors are intertwined with mental health and play a critical role in the onset, severity, and duration of various mental illnesses, including depression (Kochhar et al., 2020). During periods of quarantine, issues of food insecurity are intensified; food availability is limited; nutrition quality suffers; and, people often go without meals to preserve their limited resources, either due to financial hardships or scarcity of nutritious food even when it can be afforded. One might reasonably expect that due to such factors, the pandemic would have a significant impact on the mental health of those residing in RZDs who experience even further marginalization and exacerbation of the stressors related to long-term financial, food, community, and familial insecurity as a result of extended quarantine.

While other studies infer that such circumstances will lead to greater mental health problems, we could not find any research focusing on the mental health of individuals residing in high-risk, marginalized communities in Latin America, such as the RZDs in Guatemala. For example, one study in Argentina found that despite a low number of confirmed COVID-19 cases and deaths in the country, the general population still reported elevated rates of anxiety, stress, and depression and unfavorable social and economic conditions are proposed as potential explanations for this. However, there is no further elaboration on this potential relationship and no context is provided to understand the nature of the association (Badellino et al., 2020). Further, Garcia et al. (2020) discuss mezzo and macro level issues in eight countries across Latin American and for the region as a whole, but do not connect these issues to mental health outcomes.

What is noteworthy is that these communities were dealing with ongoing crises, never fully resolved, within contexts affected by fragile safety nets and a chronic lack of resources. The pandemic, in such contexts, becomes a complex emergency (FAO, 2020), with a compounded impact on the health and mental health of these communities and the overall state of wellbeing. Yet, no studies to date explored the pandemic as a complex emergency, considering the prior socioeconomic challenges as preconditions for negative mental health outcomes of the pandemic on these populations.

This study aims to address this gap and examine the psychological effects of the COVID-19 pandemic and extended quarantine on individuals residing in highly vulnerable RZDs across and surrounding Guatemala City. We hypothesize that individuals in these communities will report significant levels of psychological distress, particularly those in multi-member households (where caregiver burden is increased), women (who are most often in the caregiver role), and those with physically ill family in their household (generating and reinforcing contagion/infection fears).

## Methods

### Sample

With the approval of the appropriate Institutional Review Board and in collaboration with Hunger Relief International and International Social Work Solutions, a total of 295 individuals from 11 districts in and around Guatemala City participated in the Covid Care Calls study (CCC). The study was conducted in partnership with local practitioners that were trained to conduct the phone interviews – within the parameters of the IRB approval secured by the principal investigators. Participants were provided with information on the nature of the study and their rights as research participants, and verbal informed consent was secured for all calls. The CCC was established in response to the global COVID-19 pandemic to address developing community needs including, communicating accurate information about COVID-19, the provision of emotional support to vulnerable individuals and families, and facilitating referrals for mental health and health care, and other resources.

The CCC study entailed making weekly telephone calls to people whom Hunger Relief International (HRI) has served in various programs in and around Guatemala and who were referred by community partners. Callers administered a semi-structured interview to elicit information regarding medical, mental health, economic, and psychosocial status, and to provide information, assistance, and appropriate referrals to vulnerable people at-risk of COVID-19 infection and experiencing other pandemic-related challenges; and recorded their own observations of subjects during the interviews. The objectives of the CCC program are to: (1) identify main challenges related to the COVID-19 pandemic, for these communities; (2) provide emotional support for people either suffering from symptoms of COVID-19 or showing psychological distress likely attributable to living through the pandemic; (3) make referrals for medical and mental health care; and (4) prevent the spread of COVID-19 by providing education on evidence-based protective measures such as social distancing, regular hand-washing, and mask-wearing. The study PIs designed the semi-structured interview, trained callers, and provided support and supervision to in-country staff. The calls are made by HRI-based social workers and psychology interns. The CCC was carried out in multiple districts in and around Guatemala City, designated as *Red Zone Districts (RZD) and* marked by high rates of poverty and violent crime and where delinquency, teenage pregnancy, gang violence, drug abuse, and domestic violence run rampant.

For this study, all surveys administered between June 6th, 2020 and September 30th were included in the analysis, for a total of 295 participants. On average, calls lasted about 14.9 minutes.

### Measures

#### Sociodemographic characteristics

Participants provided information regarding their sex, age, number of children, and number of family members in the household, and having a sick member of the household with either a diagnosis of COVID-19 or symptoms of the virus.

#### Clinical characteristics

##### Anxiety

Participants were asked to respond yes or no to the question, ‘Have you been feeling anxious since the pandemic began?’ If they answered yes, they were than asked two follow-up questions including, ‘On a scale of 1 to 10, 1 being the lowest and 10 being the highest, how anxious do you feel?’ and, ‘Can you share with me the reasons for your anxiety?’

##### Depression

Participants were asked to respond yes or no to the question, ‘Have you been feeling depressed since the pandemic began?’ If they answered yes, they were than asked two follow-up questions including, ‘On a scale of 1 to 10, 1 being the lowest and 10 being the highest, how depressed do you feel?’ and, ‘Can you share with me the reasons for your depression?’

##### Stress

Participants were asked to respond yes or no to the question, ‘Have you been feeling stressed since the pandemic began?’ If they answered yes, they were than asked two follow-up questions including, ‘On a scale of 1 to 10, 1 being the lowest and 10 being the highest, how stressed do you feel?’ and, ‘Can you share with me the reasons for your stress?’

##### Burnout/fatigue

Participants were asked to respond yes or no to the question, ‘Have you been feeling burned-out or fatigued since the pandemic began?’ If they answered yes, they were than asked two follow-up questions including, ‘On a scale of 1 to 10, 1 being the lowest and 10 being the highest, how burned-out or fatigued do you feel?’ and, ‘Can you share with me the reasons for your burnout/fatigue?’

##### Sense of safety

Participants were asked about their sense of safety at home. They were asked to respond yes or no to the question, ‘Do you feel that it is dangerous for you or your loved ones to remain at home?’ If the participant responded yes, a follow-up question was asked, ‘Have there been any physical, verbal, or other assaults in the home during the quarantine period?’ The caller would then list other household members and ask if that person was the perpetrator to avoid the participant having to say the name and potentially raise the suspicion of someone listening nearby. Legal and safety resources were then provided to the participant and they were asked if they would like to be called in 2 days for a safety follow-up. Interviewers’ notes/observations represented an added source of information for contextualizing the answers to these questions.

Lastly, participants were asked to share any other feelings they are experiencing that they might have been asked about. More specifically, participants were asked, ‘Is there anything else you are experiencing or feeling that you would like to discuss?’ For those who responded yes, they were then invited to share what they were experiencing.

### Data analysis

Descriptive statistics were computed for the sociodemographic characteristics of the sample and the study variables consisting of frequencies and percentages for categorical variables and means and standard deviations (*SD*s) for scale variables using *t*-tests and chi-square as appropriate. Separate binary logistic regressions were estimated to examine the association between sociodemographic variables (age, sex, number of children, number of household members, having a sick household member) and each of the mental health symptoms (anxiety, stress, burnout, depression, escalation of pre-existing mental health symptoms, and sense of safety at home).

All the tests were two-tailed, with a significance level of p<0.05. The statistical analyses were performed using IBM SPSS Statistics for Windows, version 27 (IBM Corp., USA).

## Results

Participants were largely 64.3 % female, with an average age of 35 (±15.9). On average there were 4.5 people living in a household (±2.4). In terms of mental health symptoms, 64% of the sample reported symptoms of anxiety, 47% stress, 25% exacerbation of pre-existing mental health conditions, 19% depression, 18% burnout, and 5% concerns about their safety at home.

Results of the bivariate analyses comparing individuals who reported negative mental health symptoms (anxiety, depression, stress, burnout, exacerbation in pre-existing mental health symptoms, and being unsafe at home) as a result of the pandemic and those who did not are presented in Table 1. Groups were compared on sex, age, number of children, number of people living in the household relatives, and having someone sick in the household. Results indicate significant gender-based differences on anxiety reports, with more women reporting anxiety than men (χ^2^ = 4.259, *p* = .039). Significant gender differences were also found on depression, with more women than men reporting depression (χ^2^ = 5.271, *p* = .022). Significant differences were also found for those reporting an increase in pre-existing mental health symptoms and those not reporting any increase in terms of number of children, with a greater number of children associated with an increase in symptoms (*t* = 2.066, *df* = 221, *p* = .040). Lastly, significant differences were also found between those reporting anxiety and those not reporting anxiety in terms of number of children, with a greater number of children associated with endorsing anxiety (*t* = 2.018, *df* = 230, *p* = .045). No other significant differences were found for any of the mental health variables on any of the sociodemographic characteristics.

**Table 1.** Bivariate Analysis of Mental Health Symptoms.

Socio demographic characteristic and mental health symptom	Reported MH condition (*n*)	Not reported MH condition (*n*)	Chi-square or *t*-value	*df*	*p*-Value
% (*n*)					
Anxiety					
Sex					
Female	89	58	4.259	1	.039
Male	67	24			
Anyone sick at the time of the interview					
Yes	19	127	0.190	1	.663
No	8	192			
Mean (±*SD*)					
Age	36.67 (16.136)	31.32 (14.129)	2.369	217	.019
Number of people living in the house	4.59 (2.349)	4.31 (2.611)	0.824	231	.411
Number of children living in the house	1.31 (1.484)	.93 (1.181)	2.161	197.410	.032
Stress					
Sex					
Female	74	72	0.073	1	.787
Male	43	45			
Anyone sick at the time of the interview					
Yes	11	102	1.729	1	.188
No	16	86			
Mean (±*SD*)					
Age	33.81 (14.069)	35.89 (17.003)	−0.973	198.553	.332
Number of people living in the house	4.54 (2.000)	4.40 (2.816)	0.443	227	.658
Number of children living in the house	1.36 (1.370)	1.01 (1.405)	1.927	226	.055
Depression					
Sex					
Female	22	123	5.271	1	.022
Male	24	63			
Anyone sick at the time of the interview					
Yes	4	39	0.554	1	.457
No	23	147			
Mean (±*SD*)					
Age	33.83 (16.018)	35.24 (15.451)	−0.545	211	.586
Number of people living in the house	4.70 (2.043)	4.40 (2.549)	0.721	225	.472
Mean (±*SD*)					
Number of children living in the house	1.52 (1.502)	1.11 (1.364)	1.809	224	.072
Burnout					
Sex					
Female	22	123	2.895	1	.089
Male	21	66			
Anyone sick at the time of the interview					
Yes	2	41	3.134	1	.077
No	25	145			
Mean (±*SD*)					
Age	35.60 (14.825)	34.76 (15.764)	0.316	211	.752
Number of people living in the house	4.74 (2.094)	4.40 (2.531)	0.835	225	.404
Number of children living in the house	1.67 (1.476)	1.08 (1.361)	2.551	224	.011
Sense of safety					
Sex					
Female	11	141	3.088	1	.079
Male	2	93			
Anyone sick at the time of the interview					
Yes	1	11	1.000		.570
No	26	189			
Mean (±*SD*)					
Age	25.50 (12.011)	35.38 (15.931)	−2.113	226	.036
Number of people living in the house	5.00 (2.582)	4.46 (2.414)	0.787	239	.432
Number of children living in the house	1.31 (1.316)	1.17 (1.417)	0.348	238	.728
Pre-existing symptoms					
Sex					
Female	38	103	0.003	1	.957
Male	24	64			
Anyone sick at the time of the interview					
Yes	4	54	2.222	1	.136
No	22	130			
Mean (±*SD*)					
Age	31.86 (14.819)	35.89 (15.988)	−1.666	209	.097
Number of people living in the house	4.74 (2.103)	4.32 (2.555)	1.155	222	.249
Number of children living in the house	1.50 (1.523)	1.07 (1.347)	1.956	99.822	.053

Table 2 presents the results of the binary regressions. All sociodemographic characteristics were retained in the models as in addition to the findings of our bivariate analyses, previous studies have found a significant association between mental health impairment and these characteristics providing a strong rationale to include them in the analyses.

**Table 2.** Binary Logistic Regressions Predicting Mental Health Outcomes.

Anxiety	Model χ^2^	95% CI	*p*-Value
	12.747		.026
	OR		*p*-Value
Sex	0.485	0.246–0.958	.011
Age	0.969	0.946–0.993	.037
Anyone sick in household	0.995	0.395–2.507	.992
Number of household members	0.957	0.794–1.154	.647
Number of children in the home	0.863	0.633–1.175	.348
Burnout	Model χ^2^	95% CI	*p*-Value
	13.509		.019
	OR		*p*-Value
Sex	0.547	0.268–1.118	.098
Age	0.995	0.972–1.018	.660
Anyone sick in household	0.274	0.061–1.229	.091
Number of household members	1.124	0.904–1.297	.294
Number of children in the home	0.664	0.464–0.949	.025
Stress	Model χ^2^	95% CI	*p*-Value
	5.115		.402
	OR		*p*-Value
Sex	1.062	0.585–1.931	.842
Age	1.004	0.985–1.024	.671
Anyone sick in household	0.587	0.251–1.371	.218
Number of household members	1.088	0.914–1.295	.342
Number of children in the home	0.778	0.583–1.039	.089
Depression	Model χ^2^	95% CI	*p*-Value
	10.437		.064
	OR		*p*-Value
Sex	0.428	0.211–0.871	.019
Age	1.005	0.982–1.029	.652
Anyone sick in household	0.662	0.210–2.087	.481
Number of household members	1.045	0.847–1.289	.681
Number of children in the home	0.742	0.525–1.048	.090
Sense of safety	Model χ^2^	95% CI	*p*-Value
	8.387		.136
	OR		*p*-Value
Sex	4.636	0.888–24.186	.069
Age	1.086	1.004–1.137	.038
Anyone sick in household	0.757	0.088–6.480	.799
Number of household members	1.1.02	0.776–1.565	.586
Number of children in the home	0.899	0.509–1.591	.716
Pre-existing mental health symptoms	Model χ^2^	95% CI	*p*-Value
	6.471		.263
	OR		*p*-Value
Sex	0.929	0.480–1.800	.827
Age	1.013	0.991–1.035	.263
Anyone sick in household	0.447	0.144–1.389	.164
Number of household members	0.987	0.817–1.192	.888
Number of children in the home	0.849	0.624–1.157	.300

Results of the binary logistic regression examining anxiety indicate that the overall model was significant (χ^2^ = 12.747; *p* = .026). Individuals reporting anxiety were 10% more likely to be older (*B* = .969; *p* = .011) and approximately 50% more likely to be female (*B* = .485; *p* = .037), respectively, than those not reporting anxiety. However, the model accounted for only approximately 9% of the variance in distinguishing between those individuals with and without anxiety (Nagelkerke *R*^2^ = .089).

Results of the binary logistic regression examining burnout indicate that the model was significant (χ^2^ = 13.509; *p* = .013). Individuals with a greater number of children were 40% more likely to report burnout than those with fewer children (*B* = .664; *p* = .025). The model accounted for only approximately 11% of the variance in distinguishing between those individuals with and without anxiety symptoms (Nagelkerke *R*^2^ = .105).

No other significant results were found for anxiety or burnout. Additionally, the regression models for depression, stress, pre-existing mental health conditions, and sense of safety were not significant.

## Discussion

This is the first study to examine the sociodemographic factors associated with the experience of mental health impairment during quarantine related to the COVID-19 pandemic among individuals in high-risk, marginalized communities in Guatemala. We found that based on self-reported experiences, a concerning number of individuals are experiencing significant negative health outcomes associated with the pandemic especially in terms of anxiety, stress, and an exacerbation of pre-existing mental health symptoms. This is the first study to examine and identify the mental health impact of the COVID-19 pandemic in Guatemala.

We also found that those individuals experiencing anxiety were significantly more likely to be older and more likely to be female than those not reporting anxiety, and that those reporting burnout were significantly more likely to have a greater number of children than those not reporting burnout.

Several important issues need to be addressed, in relation to these findings, starting with the importance of gender in assessing the mental health impact of the pandemic in highly vulnerable communities. The existing literature indicates that the burden of care is disproportionately higher for women (Raygada & Mendoza, 2020; UN, 2020), and in highly vulnerable, low income communities with little resources and a very precarious safety net, such burden is exacerbated by any additional crisis (Piras, 2020). The mezzo level pre-existing conditions increase the perception of risk for women, with the rise in domestic violence (Lopez-Calva, 2020; The World Bank, 2020b; UN Women, 2020), increased exposure to the virus, loss of income, and additional caregiving responsibilities (UN, 2020) compounding to transform a public health crisis into a complex emergency (FAO, 2020; Plomecka et al., 2020). School closings, in the absence of other child care support services, further increased women’s responsibilities (UN, 2020; UN Women, 2020), contributing to higher anxiety, while families with a greater number of children experienced significant burnout, yet another characteristic of the complex emergency the pandemic represents for these communities.

Second, the importance of age, in relation to coping, resilience, and ability to respond to complex emergencies in general, and the COVID-19 pandemic in particular, needs to be acknowledged. During this pandemic, the older population seemed to be the most vulnerable (Koma et al., 2020; WHO, 2020); and it is definitely further compounded by pre-existing vulnerabilities such as job insecurity; higher dependence on other family members; increased sense of hopelessness; and high levels of anxiety regarding the wellbeing of children and other family members. For older men, existing research suggests that the loss of capacity and the increased health and mental health vulnerabilities, combined with their reluctance to seek medical help contribute to higher levels of anxiety and stress, and an overall deterioration of mental health (Mastroianni, 2020). For older women, caregiving roles, the sense of responsibility for their families, compounded with their increased vulnerabilities (and in the case of the pandemic, the increased risk for contagion and death) also contribute to a rise in anxiety and depression (Tandon & Meeta, 2020; UN Women, 2020). Additionally, the strict mitigation measures implemented during this pandemic, leading to increased isolation of older adults, further exacerbate the stress and anxiety amongst this population (Banerjee & Rai, 2020; Losada-Baltar et al., 2020), particularly in the context of communities that rely on social networks of care.

We did not find any significant differences between those reporting stress, depression, increased pre-existing mental health symptoms, or sense of safety and those not reporting these symptoms in terms of age, sex, number of children, number of people in the household, or having a sick household member with either a diagnosis of COVID-19 or symptoms of the virus.

The absence of significance in and of itself raises a red flag, as these communities were already experiencing higher levels of community and domestic violence, only to be exacerbated by the pandemic (Lopez-Calva, 2020), yet the number of people identifying such issues during the calls was surprisingly low. Normalization of violence (Plata, 2018), as well as the stigma attached lead us to conclude that further exploration of these issues is necessary. Is the pandemic taking precedence, and becoming the acute crisis people need to focus on – leading to minimizing or overlooking other pre-existing challenges? And, what will be the long-term impact of the current complex emergency on the mental health and overall sense of safety and wellbeing, for these communities, in the absence of targeted interventions addressing the preconditions that are yet to be explored and addressed?

Similar questions apply to understanding the somewhat generalized sense of anxiety, and the possibility of depression being masked by or misidentified as anxiety. Interestingly enough, in our study women were more likely to identify depression, or depression related symptoms than men, yet depression in general remained underreported. It is not clear if the underreporting was due confusions on the terms used in the questions, or to a normalization of depression, or to a self-conscious choice of keeping it private, as all of these factors could contribute to under-recognition and underreporting of symptoms. Further explorations of these self-identified mental health issues and the degree of normalization, sense of helplessness, or stigma that might affect people’s reports on such issues will shed more light on immediate and long-term implications of complex emergencies in general, and the COVID-19 pandemic in particular, for low income, highly vulnerable communities.

### Limitations

The findings should be considered in the context of several methodological limitations of the study. First, we focused on individuals residing in high-risk, marginalized communities in a low-income country. They, therefore, may not be generalizable to high risk, low-income communities within middle or high-income countries or to higher SES communities as their mental health profile may differ. However, some important questions raised by this study, should be considered and explored in highly vulnerable communities across countries, to identify both commonalities and differences, particularly in relation to the contribution of mezzo factors on the impact of the pandemic on mental health. Second, the study did not utilize a longitudinal design and therefore cannot speak to changes in mental health status over time. We are unable to determine if impairments in mental health functioning improved, worsened, or remained the same throughout the quarantine period. Future research should examine the long-term mental health impact of the pandemic on mental health during the course of the quarantine as well as post-quarantine periods, with particular attention to the pre-existing social and economic challenges faced by these communities. Third, although mental health functioning was assessed using standard clinical language (depression, anxiety, stress, and burnout), it may be the case that more culturally relevant terms would have yielded greater endorsement of symptoms. However, interviewers were trained to ask follow-up questions regarding common symptoms associated with each condition and to explain the terms to participants using culturally-grounded descriptions when clarification was needed. Nevertheless, follow-up studies should be conducted using alternate terms or culturally bound expressions of illness to improve accuracy in identifying depression, anxiety, stress, and burnout.

## Conclusion

Based on our study, individuals residing in high-risk communities characterized by pre-existing extreme poverty, food insecurity, and high rates of crime, gang activity, teenage pregnancy, substance abuse, and domestic and familial violence are experiencing alarming rates of psychological distress related to the current pandemic. For such communities, the pandemic needs to be reframed as a complex emergency, with the pre-existing socioeconomic challenges being explored as pre-existing conditions, contributing to a higher impact of the pandemic on the health and mental health of these communities. Such communities are affected by an overall fragile and precarious safety net, more specifically a weak mental health care system, and little to no resources allocated to the highly vulnerable communities that need them the most. Within these communities, the vulnerability levels are even higher for older adults, women, and families with greater number of children in their care. Older adults and women are particularly vulnerable to anxiety and burnout. While not surprising, due to the extensive literature talking about the disproportionate burden of care for women (Bando, 2019), it is important to think of the pre-existing socioeconomic conditions as well as the lack of protection for women as caregivers as contributing to these higher rates in the context of the COVID-19 pandemic. In these marginalized communities where resources for mental healthcare are lacking, the safety net is precarious at best, and the presence of disease, ongoing stress, and a low sense of safety is the norm, it is important to change the discourse on pre-existing conditions, and include prior socioeconomic challenges in any further explorations of the impact of the pandemic on mental health. This could lead to targeted interventions, allowing us to maximize resources by strengthening informal networks of care, while at the same time creating a framework for action leading to policy changes at local and national levels in regards to the allocation of resources needed to mitigate and contain the spread of the virus and to prevent escalation of mental health issues over time, in highly vulnerable communities. Interdisciplinary approaches, engaging humanitarian assistants (to understand complex emergencies), psychologists and other mental health care professionals (to frame mental health care interventions), and social workers (for multilevel systemic responses to complex emergencies, in highly vulnerable communities) are recommended, to develop innovative responses to the COVID-19 pandemic and its yet to be fully known impact over time.
